# High-Complexity Questions and Their Answers for Everyday Heart Failure

**DOI:** 10.3390/jcm14113993

**Published:** 2025-06-05

**Authors:** Amelia Campos-Saénz de Santamaría, Javier Pérez-Santana, François Croset, Laura Karla Esterellas-Sánchez, Victoria Lobo-Antuña, Miriam Ripoll-Martínez, Sofia Russo-Botero, Henar Gómez-Sacristán, José Pérez-Silvestre, José María Fernández-Rodriguez, Marta Sánchez-Marteles, Prado Salamanca-Bautista, Jorge Rubio-Gracia

**Affiliations:** 1 University Clinical Hospital of Zaragoza, 50009 Zaragoza, Spainjorgerubiogracia@gmail.com (J.R.-G.); 2 Aragon Health Research Institute (IISA), 50009 Zaragoza, Spain; 3 University Hospital Nuestra Señora de la Candelaria, 38010 Tenerife, Spain; 4 University Hospital Ramón y Cajal, 28034 Madrid, Spain; 5 University General Hospital Consortium of Valencia, 46014 Valencia, Spain; 6 University Hospital Puerta de Hierro Majadahonda, 28034 Madrid, Spain; 7 Central University Hospital of Asturias, 33011 Oviedo, Spain; 8 University Hospital Virgen Macarena, 41009 Seville, Spain

**Keywords:** AF: atrial fibrillation, AHF: acute heart failure, CA125: carbohydrate antigen 125, CKD: chronic kidney disease, eGFR: estimated glomerular filtration rate, LVEF: left ventricular ejection fraction, NT-proBNP: N-terminal pro-brain natriuretic peptide, VExUS: venous excess ultrasound protocol

## Abstract

As part of the “2nd Training Conference on Heart Failure and Atrial Fibrillation for Residents”, held in Madrid in November 2024, a collaborative initiative was launched to address the most common practical challenges in the management of heart failure (HF) in daily practice. This document is the result of the joint efforts of residents from various hospitals nationwide, in collaboration with senior physicians with extensive HF expertise and members of the Working Group of the Spanish Society of Internal Medicine. Our aim is to provide a useful tool that promotes learning and collaboration among professionals interested in this field. The structure of this document is based on a compilation of the most interesting and challenging questions raised during the conference. Each question is addressed with a concise and practical response, supported by updated references to ensure scientific rigor and facilitate consultation.

## 1. Introduction

Heart failure (HF) represents a growing global health challenge, with an increasing prevalence and a significant impact on morbidity, mortality, and healthcare resources. Advances in pharmacological and non-pharmacological treatments have led to improved outcomes, yet the complexity of HF management continues to pose challenges for clinicians. Multidisciplinary approaches are essential for optimizing patient care, ensuring timely interventions, and integrating novel therapeutic strategies. However, there remain several high-complexity clinical scenarios where evidence-based guidance is lacking, necessitating ongoing discussion and expert consensus.

This document seeks to address these critical uncertainties by analyzing complex clinical questions encountered in everyday HF management. By providing clear, practical, and up-to-date answers, it serves as a valuable resource for clinicians striving to deliver the best possible care in this rapidly evolving field.

## 2. Materials and Methods

The questions analyzed in this document originated from discussions held during the “2nd Training Conference on Heart Failure and Atrial Fibrillation for Residents”, which took place in Madrid in November 2024. A total of ten key questions were selected based on their clinical relevance and the challenges they present in daily practice. Special attention was given to selecting questions in which clinical practice guidelines are often unclear, where there is no established consensus, or where a scientific evidence gap currently exists. Their selection, analysis, and elaboration were carried out collaboratively by national HF experts and residents from various hospitals across the country. Each response was supported by high-quality, up-to-date scientific evidence, with appropriate references provided to ensure rigor and facilitate further consultation. This multidisciplinary effort aimed to offer a comprehensive and practical resource addressing key challenges in everyday clinical practice.

Despite the progress made in recent years, several areas in HF management—especially regarding complex clinical scenarios—remain underexplored and require future studies with higher-quality evidence to strengthen and refine current practices.

## 3. Ten Complex Key Questions About HF

### 3.1. Management of Recovered or Improved LVEF: Should Neurohormonal Treatment Be Continued?

Neurohormonal therapy for patients with reduced ejection fractions includes angiotensin receptor–neprilysin inhibitors (ARNIs), beta-blockers, and mineralocorticoid receptor antagonists (MRAs). In patients with improved or recovered LVEF, neurohormonal treatment should be maintained [1]. Although an improvement in LVEF is a positive sign, these patients remain at risk of HF progression. Neurohormonal therapies play a crucial role in maintaining clinical stability, preventing the recurrence of left ventricular dysfunction, and reducing the long-term risk of adverse events.

These recommendations are supported by the TRED-HF [2] clinical trial, which demonstrated that discontinuing pharmacological HF treatment in patients with recovered dilated cardiomyopathy resulted in a 40% relapse rate.

### 3.2. Mineralocorticoid Receptor Antagonists: Evidence and Dosage in Preserved Ejection Fraction HF

For patients with HFpEF who are already on the optimal therapy with diuretics and SGLT2i (+ semaglutide/tirzepatide if BMI > 30 kg/m^2^) and still exhibit symptoms (NYHA II-IV), the initiation of MRAs may be considered if K+ < 4.7 mmol/L and the eGFR > 30 mL/min (for finerenone: ≥25 mL/min) [3]. The dosage guidelines are as follows:

- Spironolactone: An initial dose of 12.5 mg/24 h, targeting 25–50 mg/24 h, provided there are no limitations due to hyperkalemia, renal impairment, or hypotension.
- Eplerenone: An initial dose of 25 mg/24 h, targeting 50 mg/24 h as tolerated.
- Finerenone: An initial dose of 20 mg/24 h, increased to 40 mg/24 h if the eGFR > 60 mL/min. If the eGFR is lower, start at 10 mg/24 h, increasing to 20 mg/24 h if tolerated.

Key differences between spironolactone and eplerenone [4]: Eplerenone was synthesized to avoid the anti-androgenic effects of spironolactone (e.g., gynecomastia, impotence, breast tenderness). Both medications share common side effects like hyperkalemia and an initial reduction in eGFR. No double-blind randomized clinical trials directly compare them in HF, but real-world data suggest that eplerenone may offer better cardiovascular outcomes.

The evidence for this is primarily provided by two clinical trials [4]:

- TOPCAT [3]: A clinical trial in which patients with symptomatic HF and LVEF of 45% or higher were randomly assigned to spironolactone vs. placebo. The study did not show statistical significance regarding mortality, but it did show significance for hospitalization due to HF. A subsequent subgroup analysis demonstrated statistical significance in the primary outcome (a combined event of cardiovascular death and hospitalization for HF) in the North American and Western European groups, but not in the Eastern European group. It also showed greater efficacy in patients with a LVEF < 60% and in women, where the reduction in events was similar across all EF ranges.
- FINEARTS [5,6,7]: The recent FINEARTS-HF study evaluated the effect of finerenone in HF with preserved or mildly reduced EF (EF ≥ 40%). Finerenone, compared to placebo, reduced the occurrence of the combined event of cardiovascular death and HF hospitalization. The dose was adjusted according to the patients’ eGFR according to the guidelines and the product label: → For patients with an eGFR > 60 mL/min/1.73 m^2^, treatment started with 20 mg daily, which could be increased to 40 mg if no adverse effects occurred. → For patients with an eGFR ≤ 60 mL/min/1.73 m^2^, treatment started with 10 mg daily, which could be increased to 20 mg if well tolerated. → For an eGFR < 25 mL/min/1.73 m^2^, the treatment was contraindicated. → This dosing regimen aimed to minimize the risk of hyperkalemia. The target dose was 20 mg daily, adjusted based on serum potassium levels after 4 weeks.

**Figure 1:**
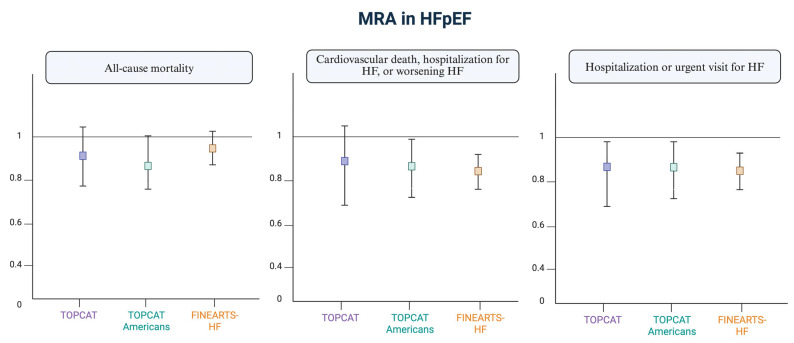
Current evidence on MRAs in patients with preserved ejection fraction. Adapted from “Cannata A and McDonagh TA. Heart Failure with Preserved Ejection Fraction. N Engl J Med. 2025” [10]. Figure created with Biorender.

Outside the scope of HF, CKD and type 2 diabetes, finerenone has already demonstrated a reduction in the risk of CKD progression and a decrease in cardiovascular events, including HF hospitalization (placebo-controlled clinical trials IDELIO-DKD and FIGARO-DKD) [8,9]. Its indication is for CKD with a filtration rate >25 mL/min in patients with diabetes, significant albuminuria, and those receiving optimized treatment with renin–angiotensin system antagonists. Figure 1 [10] summarizes the main findings of MRAs in heart failure with preserved ejection fraction (HFpEF).

### 3.3. The Sequential Blockade of the Nephron: Permanent or Intermittent? What Are the Differences Between Hydrochlorothiazide and Chlorthalidone?

**Figure 2:**
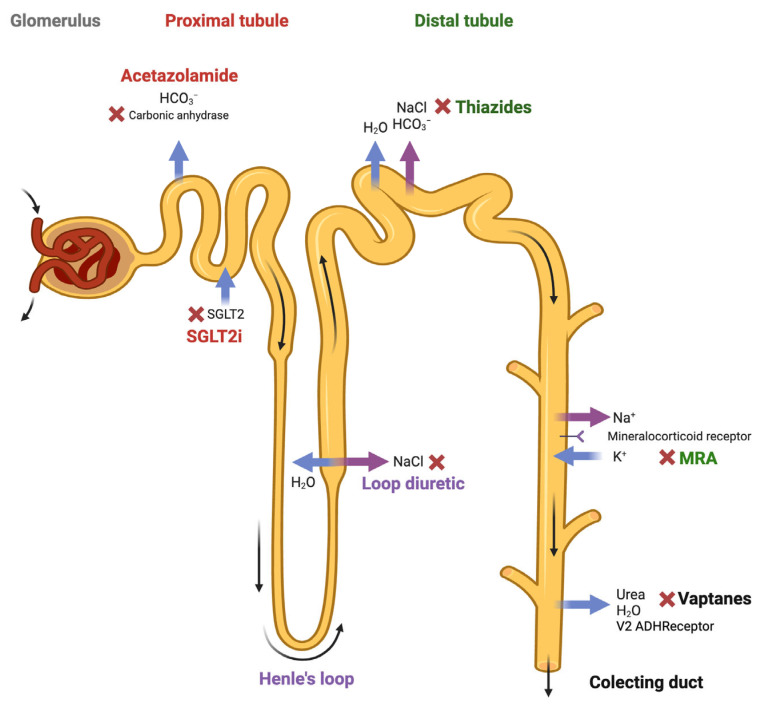
The possible diuretic mechanisms of action in the sequential blockade strategy of the nephron. Figure created with Biorender.

The sequential blockade of the nephron is a therapeutic strategy used in patients with an altered sensitivity to diuretics, which limits their ability to reach euvolemia. The academic definition of diuretic resistance is not clearly established; it usually refers to when patients need to take two or more doses of loop diuretics per day or those who do not achieve adequate natriuresis (<50 mEq/L) with sufficient diuretic doses. This strategy involves combining different types of diuretics that act at distinct segments of the nephron. The idea is to complement the mechanisms of action of these drugs, especially when the effect of a single agent in monotherapy is insufficient. Figure 2 shows the different possible diuretic mechanisms of action in the sequential blockade of the nephron. The consensus document recommends combining hydrochlorothiazide or acetazolamide if, after the second increase in intravenous furosemide, the patient still has an insufficient diuretic response [11].

Although there is no definitive rule for deciding when to block the nephron intermittently or permanently, some recommendations have been made: When we encounter patients who respond well to a single loop diuretic, sequential blockade is not considered. However, in patients who do not respond well to high doses of furosemide/torasemide and persist with congestive symptoms, we propose a sequential blockade [12]. These will generally be patients with diuretic resistance, associated CKD, and/or persistent congestion. Both options are correct and possible. The decision should be individualized based on the patient’s clinical and analytical situation, and primarily on the specific therapeutic goal intended to be achieved with that patient.

The CLOROTIC trial [13] was the first randomized, multicenter, double-blind clinical trial that evaluated the efficacy and safety of adding a thiazide diuretic in chronic arterial congestion. In this trial, patients took home doses of furosemide between 80 and 240 mg, and the thiazide diuretic used was hydrochlorothiazide; thus, there are no data on the use of chlorthalidone. Both hydrochlorothiazide and chlorthalidone have bioavailabilities of approximately 60–80% and are primarily excreted renally. Chlorthalidone has a longer half-life of 40–60 h compared to that of less than 24 h for hydrochlorothiazide. Hydrochlorothiazide reaches its maximum plasma concentration between 1 and 5 h, beginning its diuretic action at 2 h and with maximum effects at 4 h. In contrast, chlorthalidone reaches its maximum plasma concentration after 8–12 h. The benefit of chlorthalidone from this perspective is fundamentally dosage-related; when administered at night, the maximum plasma concentration is reached the next morning, coinciding with the administration of furosemide and thus achieving a greater diuretic response. On the other hand, it must be considered that chlorthalidone has a higher risk of hypotension and electrolyte disturbances.

### 3.4. Hypertonic Saline: Evidence and Dosage

Hypertonic saline therapy in HF with persistent congestion can be beneficial, particularly in patients with diuretic resistance and predominant tissue congestion. Its primary mechanism of action consists of increasing sodium delivery to the renal tubules, enhancing the osmotic effect, and replenishing the intravascular compartment, which improves the efficacy of loop diuretics.

Initially, the SMAC study [14] concluded that the use of hypertonic saline combined with high-dose furosemide could reduce hospital stays and mortality. Since then, studies have shown that the combination of hypertonic saline with furosemide improves several clinical parameters, such as increased diuresis, weight reduction, and shorter hospital stays compared to the use of furosemide alone. However, evidence on its impact on mortality and rehospitalizations remains insufficient and highly variable, highlighting the need for larger and more robust trials.

According to the Working Group of the Spanish Society of Internal Medicine and the latest consensus on diuretics, hypertonic saline therapy is recommended when there is failure of sequential nephron blockade and persistent congestion [15]. In particular, there is one clinical scenario where its use can be especially effective: patients with manifest congestion and hypochloremic hyponatremia.

**Figure 3:**
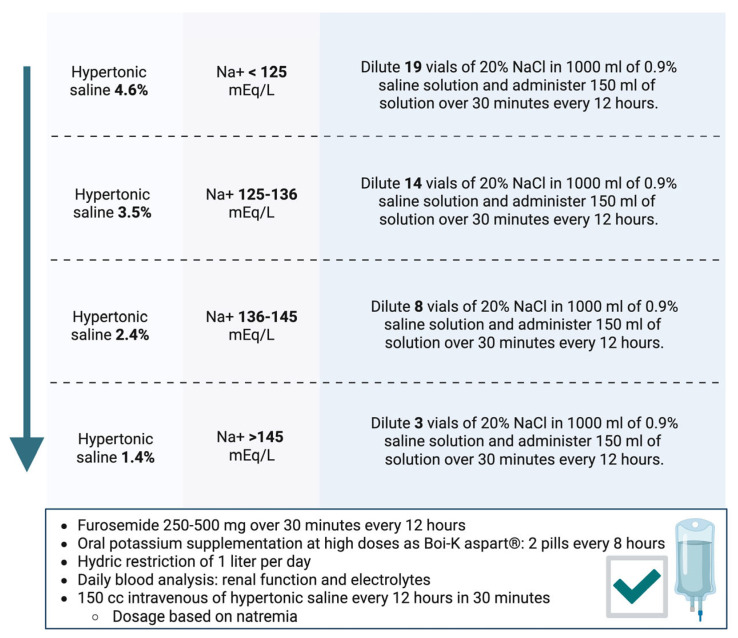
Hypertonic saline protocol based on natremia developed by Spanish Society of Internal Medicine [16].

Our working group developed a document on the dosing of hypertonic saline based on natremia, indicating a different dilution of 20% saline ampoules in 0.9% saline, as shown in Figure 3 [16]. After dilution, the dosage is as follows: 150 mL of the dilution along with high-dose furosemide (250–500 mg) administered via an intravenous infusion pump over 30–60 min every 12 h. Furthermore, hypertonic saline administration requires high-dose potassium supplementation and close analytical monitoring to watch for ionic imbalances.

The recommended infusion time is variable, although experts suggest maintaining it for at least three days. However, different healthcare settings (hospital ward, day hospital, HF unit…) may adjust the duration based on the patient’s profile and available resources [17].

### 3.5. In a Cardiorenal Setting and in Patients with Low Glomerular Filtration, Should Disease-Modifying Drugs Be Discontinued?

Updated evidence highlights the nephroprotective role of certain disease-modifying drugs. Recent trials have shown that SGLT2 inhibitors significantly slow kidney function decline in patients with CKD, both with and without type 2 diabetes. For instance, in patients with albuminuric CKD, SGLT2i [18,19] or finerenone [20] use on top of standard therapy reduces the annual eGFR decline to 2–2.5 mL/min/1.73 m^2^, and in normoalbuminuric CKD, SGLT2i reduces this loss to <0.5 mL/min/1.73 m^2^—well below the typical age-related decline. These findings support the continued use of these therapies, even in patients with advanced CKD, emphasizing their role not only in cardiovascular protection but also in preserving renal function.

However, sometimes there are limited data to support recommendations on the treatment of patients with HF and severe CKD, as clinical trials to date have excluded patients with advanced-stage CKD. Regarding ACEi and ARBs, their prescribing information allows for their use down to an eGFR of <15 mL/min/1.73 m^2^, even though there are studies that support their use during dialysis due to their antiproteinuric effect [20]. Recently, the use of ARNIs in HF patients undergoing hemodialysis has also been studied, showing improvements in LVEF and reduced mortality [18]. It is important to note that, in pivotal trials, the cutoff values for patient inclusion were 25 mL/min/1.73 m^2^ in the DAPA-CKD study [19], 20 mL/min/1.73 m^2^ in EMPEROR-Reduced [21] and GALACTIC-HF [22], and 15 mL/min/1.73 m^2^ in the VICTORIA study [23]. In HFrEF patients who develop renal insufficiency due to cardiorenal syndrome, the decision to discontinue disease-modifying drugs should be individualized. The first step should be to assess the cause of renal function decline, considering the following: low cardiac output, the hemodynamic effects of medications (such as reduced renal perfusion due to efferent vasodilation induced by ACEIs, ARBs, or ARNIs), and reversible causes of renal failure, such as hypovolemia, hypotension, or nephrotoxicity from other substances.

**Figure 4:**
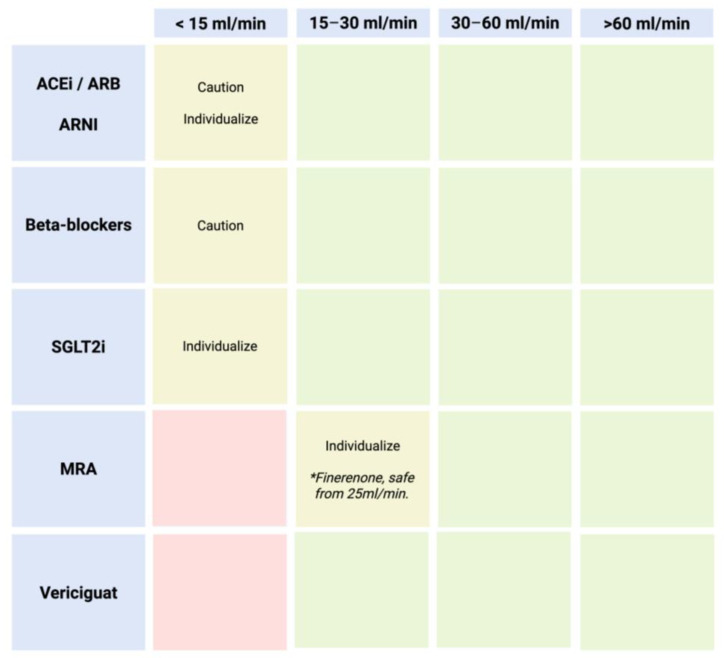
Practical diagram on safety and pharmacological experience according to glomerular filtration rate. Green: safe; yellow: caution, individualize; red: avoid. * Finerenone is the only MRA safe from 25 mL/min.

In general, key considerations regarding the decision to discontinue disease-modifying drugs include the following: (1) Withdrawal increases the risk of death or hospital readmission. (2) The maximum survival benefit is associated with sacubitril/valsartan and beta-blockers. Specifically, each medication should be regarded in the context of renal insufficiency due to cardiorenal syndrome [16,24]. Figure 4 presents a practical diagram illustrating safety considerations and pharmacological experience based on glomerular filtration rate.

- ACEi/ARBs/ARNIs: These are ideally used until an eGFR of 15 mL/min. Evidence supports their continuation even beyond this threshold on an individualized basis.
- SGLT2i: Indicated until an eGFR between 10 and 15 mL/min. For lower filtration rates, individualized treatment should be considered based on the patient’s clinical benefit.
- MRAs: These should be initiated with an eGFR ≥30 mL/min (except for finerenone: ≥25 mL/min) [25]. However, if the eGFR declines and potassium levels are within normal limits, they should be maintained, as they continue to improve prognosis. Regarding the initiation of these drugs, they are not recommended if the eGFR is below 30 mL/min/1.73 m^2^. It is important to note that this recommendation is based on the lack of clinical trials including patients with severe renal insufficiency. However, once started, a rise in serum creatinine up to 50% of baseline values is acceptable, as long as it is <266 μmol/L (3 mg/dL), and a reduction in eGFR of up to 10% from baseline is acceptable, as long as the eGFR remains >25 mL/min/1.73 m^2^.
- Beta-blockers: Similarly to other medications, there is no evidence for their use in patients with severe renal insufficiency (eGFR < 30 mL/min/1.73 m^2^). However, they should generally be maintained based on blood pressure and heart rate, as they have been shown to reduce mortality in moderate renal dysfunction (eGFR 45–59 mL/min/1.73 m^2^) as well as moderately severe renal dysfunction (eGFR 30–44 mL/min/1.73 m^2^).
- Vericiguat: Indicated until eGFR < 15 mL/min.

### 3.6. Beta-Blockers, Should They Be Discontinued in Acute Heart Failure?

The well-known negative inotropic effect of beta-blockers might suggest that it is necessary to discontinue their use in patients admitted for acute HF to avoid worsening the patients’ hemodynamics. On the other hand, some older studies have shown that the abrupt discontinuation of these medications is associated with an increased risk of myocardial infarction and arrhythmias [26].

A randomized study and several observational studies have tried to answer this question, showing a protective effect of this drug class when maintained throughout hospitalization [27,28]. Of particular interest is an observational study conducted on 1420 patients which demonstrated that maintaining beta-blockers was associated with a lower risk of death (HR 0.60, *p* = 0.04) after adjusting for variables such as blood pressure, cardiac index, and LVEF. The benefit of maintaining beta-blockers was also confirmed in patients treated with inotropes (milrinone) during hospitalization (a 34% increase in the risk of death or readmission within 60 days in patients who discontinued beta-blockers), possibly due to the antiarrhythmic effect of these drugs counteracting the proarrhythmic effect of inotropes [29].

Clinical practice guidelines recommend maintaining beta-blocker therapy in patients with HFrEF admitted for acute decompensation unless contraindications such as sustained hypotension or low cardiac output are present [30].

### 3.7. Patients with Atrial Fibrillation and Heart Failure: Should I Opt for Digoxin or Beta-Blockers?

According to the latest guidelines for managing AF in patients with HF, beta-blockers and/or digoxin are recommended as first-line drugs for heart rate control and symptom reduction, regardless of LVEF [31]. In cases where LVEF > 40%, diltiazem or verapamil can also be used. In acute management, the choice of medication will depend on the patient’s characteristics, the presence of HF, LVEF, and the hemodynamic profile. In general, for acute heart rate control, beta-blockers or diltiazem/verapamil (if LVEF > 40%) are preferred over digoxin due to their faster onset of action and dose-dependent effects. In chronic management, the choice of medications for heart rate control will depend on symptoms, comorbidities, and the possibility of adverse effects due to pharmacological interactions. Combination therapy should be considered only when strictly necessary to achieve a target heart rate.

Beta-blockers, specifically cardioselective ones, are the first-line treatment for heart rate control, largely due to their immediate effect on heart rate and their demonstrated beneficial effects in patients with HFrEF (although this benefit is uncertain in patients with preserved ejection fraction and sinus rhythm). In the following studies, the use of digoxin versus beta-blockers for managing AF is compared:

- In the clinical trial “RATE-AF” [32] (evaluation of treatment used for heart rate control in patients with symptomatic permanent AF), there were no differences between low-dose digoxin and bisoprolol in terms of quality of life scales at 6 months after initiating treatment. However, digoxin showed advantages in secondary outcomes: fewer adverse effects, fewer subjective symptoms (according to the NYHA scale), and a reduction in NT-proBNP.
- In the “ESC–EHRA EORP AF Long-Term General Registry” [33], the impact of using digoxin versus beta-blockers in AF patients was compared regarding quality of life, hospitalizations, and mortality. Higher mortality was observed with digoxin, not due to the drug itself but due to the baseline characteristics of the patients. No significant differences were found in terms of hospitalizations and quality of life.

In conclusion, to date, there is no conclusive evidence regarding the superiority of beta-blockers over digoxin (or vice versa) in managing AF in patients with HF. The decision should be guided by whether the treatment is acute or chronic, the patient’s LVEF, and individual patient characteristics, with the aim of avoiding complications, adverse effects, and drug interactions.

### 3.8. The Utility of Biomarkers for Pharmacological Titration: When Should They Be Repeated?

**Figure 5:**
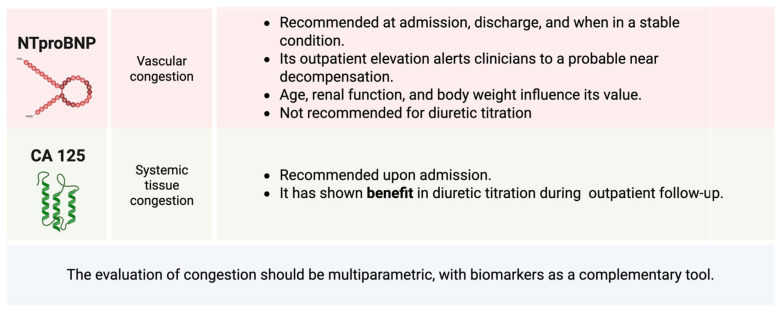
Main biomarkers in heart failure and their practical recommendations.

The N-terminal fragment of the B-type natriuretic peptide prohormone (NT-ProBNP) has proven to be a key tool for the diagnosis and prognostic stratification of HF [34]. However, NT-ProBNP has shown contradictory results regarding its use in the titration of pharmacological disease-modifying treatments. In this regard, expert groups propose practical recommendations on its use, suggesting that a new elevation or persistent significant NT-ProBNP > 1000 pg/mL during outpatient follow-up should alert clinicians to the need to optimize medical treatment, given the high risk of clinical events [35]. In these situations, it is also essential to rule out other causes of elevated NT-ProBNP, such as comorbidities or disease progression with worsening ventricular remodeling. In patients who reach an apparently stable situation with optimized pharmacological treatment, frequent serial measurements would not be necessary. It is noteworthy that taking a baseline NT-ProBNP measurement when the patient is stable is recommended, as factors such as age, BMI, or renal function can significantly influence plasma concentrations of these biomarkers [36]. This allows for more accurate knowledge of individual variations in the biomarker over time. Figure 5 illustrates the main biomarkers in heart failure along with their corresponding practical recommendations.

Regarding its usefulness in guiding the dosage of diuretic treatment, although NT-ProBNP is an indirect marker of increased filling pressures, it does not always accurately reflect the clinical congestion status. Despite multiple studies, there is no solid evidence supporting its exclusive use for titrating diuretic treatment [37]. It has been shown that a decrease in NT-ProBNP of more than 30% at discharge increases survival, and it is hypothesized that an increase in NT-ProBNP compared to discharge during outpatient follow-up reinforces the need for clinical monitoring and pharmacological optimization [38]. However, it is important to remember that congestion assessment should be based on a multiparametric approach, including clinical signs and symptoms, ultrasound findings, and biomarkers. In this way, the biomarker is integrated as a complementary tool, but not as the sole determinant in the assessment of congestion [39].

On the other hand, CA 125 has emerged as a key marker of tissue congestion and inflammation in HF [40]. However, for its correct interpretation, it is essential to consider its long half-life: 7–12 days. It is a biomarker primarily associated with systemic congestion and, therefore, with a predominance in the semiology of right-sided HF and an increased risk of adverse clinical events. Its measurement upon admission is useful in the multiparametric evaluation of congestion and establishing an appropriate phenotyping of it. One of its most attractive properties is its potential to monitor clinical course after HF decompensation and pharmacological titration.

The CA125-guided diuretic therapy clinical trial was superior to standard care in terms of mortality and readmission at one year [41]. When CA125 decreased below 35 U/mL, the protocol recommended reducing the dose of diuretics and spacing outpatient visit frequencies, while if it remained above 35 U/mL, patient follow-up frequency increased, loop diuretic use was optimized, and statin use was increased. The guided strategy was superior to standard scare in terms of reducing the combined risk of death at one year or readmission due to HF. More recently, in the IMPROVE-HF trial [42], the effect of a CA125-guided diuretic strategy (using the same cutoff as the previous study) on short-term renal parameters (in patients with renal dysfunction at the time of evaluation) was assessed, showing early improvement in clinical and renal function parameters at 72 h and a trend toward a lower risk of adverse clinical events at 30 days.

### 3.9. NTproBNP False Negatives: Does Obesity Really Influence Them?

NT-ProBNP levels, although highly sensitive markers for diagnosing HF, can present false negatives under certain circumstances. One notable case is obesity, where it has been reported that up to 20–35% of patients may have falsely low levels of BNP and ProBNP [43]. An example of this is the SUMMIT-HF study [7] with Tirzepatide, where patients with an apparently correct diagnosis of HFpEF had NT-proBNP levels of 196 pg/mL and 169 pg/mL in the Tirzepatide and placebo groups, respectively.

The etiopathogenesis of this phenomenon is still poorly understood, although possible underlying mechanisms have been identified. Among them, the increased expression of neprilysin in adipose tissue, an enzyme responsible for degrading natriuretic peptides, is prominent. Additionally, it is hypothesized that the higher plasma volume in individuals with obesity could contribute to a decrease in the relative concentrations of these biomarkers in the blood [44].

The current guidelines use a single cutoff value for NT-proBNP without considering variations associated with BMI, increasing the risk of the underdiagnosis of HF in obese patients. In this context, some studies have suggested adjusting NT-proBNP cutoff values based on the degree of obesity, aiming to optimize its clinical utility in the early diagnosis of HF in this patient group [36]. One suggested approach [34] is reducing the cutoff point by 33% for patients with a BMI of 30–34.9 kg/m^2^ and by 50% for those with a BMI ≥ 35 kg/m^2^.

This phenomenon highlights the importance of interpreting NT-ProBNP values in individual clinical contexts, considering factors such as BMI, to avoid incorrect diagnoses or delays in identifying and managing HF [45].

### 3.10. Clinical Echocardiography and Heart Failure: The Interpretation of Inferior Vena Cava Dimensions and the Role of VExUS in Patients with Suspected Pulmonary Hypertension

**Figure 6:**
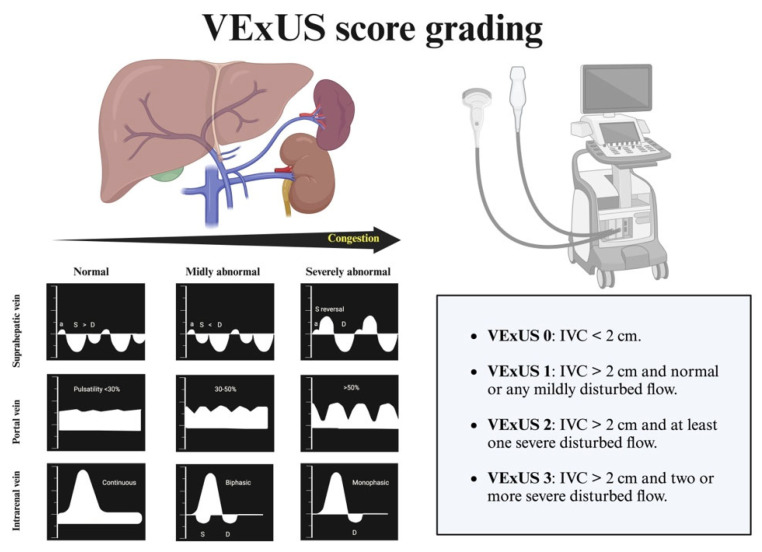
VExUS protocol.

Systemic and/or pulmonary congestion is a prognostic determinant in patients with HF. Clinical echocardiography is a key tool in quantifying congestion, allowing us to measure the IVC and apply more specific protocols such as VExUS to determine such congestion (Figure 6) [46,47]. The IVC is a highly compliant venous structure, directly anatomically related to the right atrium, and thus sensitive to pressure changes in the right atrium. An IVC diameter <2 cm and a collapsibility >50% with respiration are indicative of normal right atrial pressures. When right atrial pressure increases, it transfers blood retrogradely into the IVC, altering its size and collapsibility. There is a moderate correlation between the diameter of the IVC and its collapsibility with right atrial pressure (see Table 1) [48].

In conclusion, a dilated IVC is equivalent to increased pressure in the right atrium, either due to volume overload or increased pressure in the context of pulmonary hypertension. It is worth reiterating that there are situations, such as pulmonary hypertension, where the IVC may be dilated in the absence of vascular congestion.

The VExUS assessment aims to assess and grade the organ impairment associated with congestion [49]. This assessment begins with the examination of the IVC in its longitudinal axis, 2 cm from its entry into the right atrium. If the diameter is <2 cm and/or the collapsibility is >50%, vascular congestion is ruled out. If the diameter is >2 cm, further evaluation of the rest of the venous system is necessary. This grading system is especially useful for identifying IVC dilatations due to non-congestive causes.

For this reason, it is not recommended to rely solely on VExUS for therapeutic decision-making in patients with PH. In any case, knowing the patient’s baseline VExUS (in euvolemia) will be very helpful in identifying variations during future decompensation situations [50].

## Figures and Tables

**Table 1:** Relationship between IVC and its collapse index with central venous pressure [48].

IVC (cm)	% Collapse of IVC	Central Venous Pressure (mmHg)
<1.5	>50%	0–5
1.5–2.5	>50%	5–10
1.5–2.5	<50%	10–15
>2.5	Minimum	15–20

## Data Availability

The original contributions presented in this study are included in the article. Further inquiries can be directed to the corresponding author(s).

## Abbreviations

The following abbreviations are used in this manuscript:

ACEI	Angiotensin-converting enzyme inhibitor
AF	Atrial fibrillation
AHF	Acute heart failure
ARB	AngiotensinII receptor blocker
ARNI	Angiotensin–neprilysin receptor inhibitor
BMI	Body mass index
CA125	Carbohydrate antigen 125
CKD	Chronic kidney disease
CVRF	Cardiovascular risk factors
DM	Diabetes mellitus
eGFR	Estimated glomerular filtration rate
GLP-1 RA	Glucagon-like peptide-1 receptor agonist
HF	Heart failure
HSS	Hypertonic saline solution
IVC	Inferior vena cava
LVEF	Left ventricular ejection fraction
MRA	Mineralocorticoid receptor antagonist
mrLVEF	Moderately reduced left ventricular ejection fraction (40–50%)
NT-proBNP	N-terminal pro-brain natriuretic peptide
NYHA	New York Heart Association
pLVEF	Preserved left ventricular ejection fraction (>50%)
PH	Pulmonary hypertension
rLVEF	Reduced left ventricular ejection fraction (<40%)
SGLT2i	Sodium-glucose cotransporter-2 inhibitor
VExUS	Venous excess ultrasound protocol.
